# Predictive algorithm to stratify newborns at-risk for child undernutrition in India: Secondary analysis of the National Family Health Survey-4

**DOI:** 10.7189/jogh.12.04040

**Published:** 2022-05-14

**Authors:** Apurv Soni, Nisha Fahey, Arlene Ash, Zulfiqar Bhutta, Wenjun Li, Tiffany M Simas, Somashekhar Nimbalkar, Jeroan Allison

**Affiliations:** 1 Program in Digital Medicine, Department of Medicine, UMass Chan Medical School, Worcester, Massachusetts, USA; 2 Department of Population and Quantitative Health Sciences, UMass Chan Medical School, Worcester, Massachusetts, USA; 3 Department of Pediatrics, Bhaikaka University, Karamsad, Gujarat, India; 4 Department of Pediatrics, UMass Chan Medical School, Worcester, Massachusetts, USA; 5 Centre of Excellence in Women and Child Health, Aga Khan University, Karachi, Pakistan; 6 Centre for Global Child Health, the Hospital for Sick Children, Toronto, Canada; 7 Department of Obstetrics and Gynecology, UMass Chan Medical School, Worcester, Massachusetts, USA

## Abstract

**Background:**

India is at the epicentre of global child undernutrition. Strategies to identify at-risk populations are needed in the context of limited resources

**Methods:**

Data from children under the age of five surveyed in the 2015-2016 National Family Health Survey were used. Child undernutrition was assessed using anthropometric measurements. Predictor variables were identified from the extant literature and included if they could be measured at the time of delivery. Survey-weighted logistic regression was applied to model the outcome. Internal validation of the model was performed using 200 bootstrapped samples representing half of the total data sets.

**Results:**

In 2016, 54.4% (95% CI = 54.0%-54.8%) of Indian children were undernourished, according to a composite index of anthropometric failure. The predictive model for overall undernutrition included maternal (height, education, reproductive history, number of antenatal visits), child (sex, birthweight), and household characteristics (district of residence, caste, rural residence, toilet availability, presence of a separate kitchen). The model demonstrated reasonable discrimination ability (optimism-adjusted c = 0.67). The group of children classified in the lowest decile for risk of undernutrition had a prevalence of 25.9%, while the group classified in the highest decile had a prevalence of 77.4%.

**Conclusions:**

It is possible to stratify newborns at the time of delivery based on their risk for undernutrition in the first five years of life. The model developed by this study represents a first step in adopting a risk-score based approach for the most vulnerable population to receive services in a timely manner.

With an estimated 62 million children under the age of five experiencing stunted growth, India is at the epicentre of the child undernutrition crisis [1,2]. Although there has been a steady decline over the past two decades, a third of India’s child population experiences stunted growth and one out of five Indian children suffers from wasting [1]. Expert analyses of child undernutrition in India reveal barriers to improvement at the sociocultural and governance level [3-5]. Improving women’s status and living conditions can help address the underlying causes of child undernutrition in India [6-9]. At the same time, programs for improving proximal determinants of child growth and development, ie, access to health care, supplemental nutrition, and provision of balanced meals, are needed to prevent undernutrition among India’s vulnerable population [3-5,10-12]. India has several national-level programs that promise to improve the nutritional status of the nation’s children by 1) providing supplemental nutrition to pregnant and lactating mothers, 2) improving prenatal and infant care, and 3) addressing upstream determinants of child undernutrition ie, parental employment, access to clean water and toilet facilities, and promoting sanitary hygiene [3]. However, these programs are operated by different ministries at the national level resulting in fragmented implementation and limited accountability [3-5].

To address these governance issues, India launched a three-year ₹9046 crore (roughly equivalent to US$1.3 billion) National Nutrition Mission (NNM) in 2018 with the objective of bringing convergence across existing national-level programs. The goal of the mission is to reduce the prevalence of childhood stunting from 38.4% to 25% by 2022 [13]. A main component of the NNM is to leverage mobile technology for efficient implementation and real-time monitoring of program activities [13]. The NNM has developed a mobile application that serves as a dashboard for all nutrition-related programs. The mission also provides a mobile phone to 1.4 million frontline health workers (FHWs) to deliver services and report progress more effectively to their supervisors. The guidelines and resources for the implementation of these programs are allocated by the national government, which prioritizes 18 out of 36 states determined as high or special focus, as well as 184 out of the 640 districts classified as high priority districts [14-17]. However, a substantial burden of undernutrition persists outside of these high priority regions [18,19]. In order to empower FHWs to intervene in a timely manner and prevent child undernutrition, it is important to develop a mechanism to stratify at-risk children so that FHW’s efforts and resources can be deployed most efficiently.

Therefore, the purpose of this study was to develop and internally validate a model that could predict the outcome of child undernutrition in the first five years of life using data available at the time of delivery. We chose to limit the predictive variables to those collectable at the time of delivery, because FHWs routinely register the birth of new children within their community using the Mother and Child Tracking System (MCTS). A risk-score calculated from this data can be employed to stratify and direct focus on children at high risk of developing undernutrition.

## METHODS

### Data sources and procedures

Data from the fourth round of the National Family Health Survey (NFHS-4), 2015-2016 were used in the analyses [1]. The NFHS-4 was conducted by the International Institution for Population Sciences (IIPS), Mumbai, under the stewardship of the Ministry of Health and Family Welfare, Government of India. The protocol for NFHS-4 survey was approved by the IIPS Institutional Review Board (IRB) and reviewed by the US Centres for Disease Control and Prevention. University of Massachusetts Medical School IRB reviewed the protocol for the secondary data analyses presented in this manuscript and deemed it exempt from full review, because the data contained no personal identifiable information.

This nationally representative sample to estimate indicators at the district level (n = 640) across the 36 state and union territories was derived with a stratified two-stage sampling method using the 2011 census as the sampling frame for selection of the Primary Sampling Unit (PSUs): villages for rural stratum and census enumeration blocks in urban stratum. Within each stratum, PSUs were identified with probability proportional to size. In every selected PSU, a complete household mapping and listing operation was conducted. In the second stage, 22 households were randomly selected using systematic sampling from each PSU. This process identified 628 900 households, of which 616 346 were occupied, and 601 509 were interviewed (97.6% response rate). A total of 699 686 women aged 15-49 years responded to the women’s questionnaire and provided information on 268 873 children aged 0-59 months. Data were collected by a total of 789 field teams over a period of almost two years (January 20, 2015, to December 4, 2016). Questionnaires were administered in 17 local languages using computer assisted personal interviewing. Weight and height were measured for all children aged 0-59 months and women aged 15-49 years.

### Sample size

**Figure 1:**
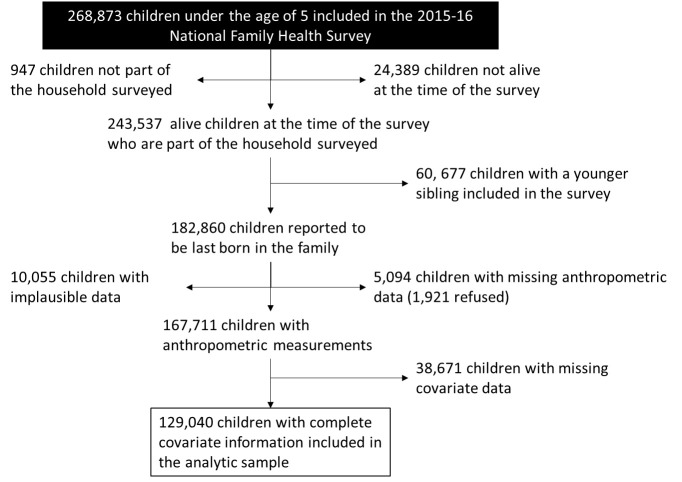
Flowchart demonstrating exclusions and final analytic sample of the 2015-16 National Family Health Survey from India included in this study.

**Figure 1** describes the derivation of the analytic sample used in this study. Children who were not alive at the time of the survey or did not belong to the household but were present at the time of survey were excluded from the analyses. Children were excluded if their younger sibling was interviewed in the survey because inclusion of multiple siblings would violate the assumption of independent observations. Of the 167 711 eligible children, data from 129 040 children were used to build a predictive model for child undernutrition due to missing or implausible data.

### Outcome variable

Child undernutrition is routinely assessed using anthropometric indicators, namely stunting (height-for-age z score<-2), underweight (weight-for-age z score<-2), and wasting (weight-for-height z score<-2). Emerging scholarship suggests that a focus on any single indicator underestimates the overall prevalence of child undernutrition. Instead, Comprehensive Index of Anthropometric Failure (CIAF), which considers a child to be undernourished if any of the three forms of undernutrition are present, is recommended [20]. Therefore we considered a child to be undernourished, if either height-for-age, weight-for-age, or weight-for-height z-score was below -2.

### **Predictor variable**s

Predictor variables were identified using the integrated framework of child undernutrition [21]. Risk factors that cannot be collected at the time of delivery, eg, breastfeeding practices, infant dietary diversity, childhood illnesses, were excluded. Additional risk factors specific to the Indian context were identified via literature review [22-24]. Maternal stature, education, ability to read local language, preceding birth interval, and age at first birth were considered. Antenatal receipt of iron supplementation for at least three months and at least four maternal visits were also considered. Child-related factors included birthweight and sex. Number of siblings, access to a toilet, rural residence, ownership of a below poverty level card, caste and religion of the household head, treatment practices of drinkable water, construction type for house, floors, and walls, use of soap for handwashing, presence of a separate kitchen, and use of non-solid fuel were the household conditions considered for the analyses. The operational definition of these variables was based on NFHS-4 guidelines and enumerated in **Table 1** [1]. The mean prevalence of CIAF per district was considered in decile groupings. Residence in high focus state and/or high priority districts was modelled using separate dummy variables.

**Table 1:** Empirical distribution and weighted proportion of predictors of child undernutrition for all eligible children with anthropometric data (n = 167 711) from the 2015-2016 National Family Health Survey of India

Variable	Overall			CIAF		
**N**	**%**	**w-%**	**n**	**%**	**w-%**
**Number of children**	167 711	100	100	89 804	53.6	54.4
**Child Factors**						
Birthweight	Missing = 36 572			Missing = 22 195		
1800 g or less	4514	3.4	3.6	3124	4.6	4.8
1801-2500 g	43 989	33.5	34.1	26 336	39.0	39.4
>2500 g	82 636	63.0	62.3	38 149	56.4	55.9
Sex	Missing = 0			Missing = 0		
Male	90 586	54.0	54.2	49 489	55.1	55.0
Female	77 125	46.0	45.8	40 315	44.9	45.0
Number of Siblings	Missing = 0			Missing = 0		
0 or 1	114 448	68.2	70.9	58 001	64.6	67.0
Two	28 957	17.3	16.4	16 602	18.5	17.8
3 or more	24 306	14.5	12.7	15201	16.9	15.2
**Maternal Factors**						
Maternal height	Missing = 404			Missing = 234		
5 feet 2 inches or taller	27 237	16.3	16.1	10 894	12.2	12.1
4 feet 8 inches to <5 feet 2 inches	105 982	63.3	63.0	55 815	62.3	61.9
<4 feet 8 inches	34 088	20.4	20.9	22 861	25.5	26.0
Maternal education	Missing = 0			Missing = 0		
No formal education	47 831	28.5	27.6	31 110	34.6	33.5
Primary (1-7 y of schooling)	36 587	21.8	21.8	20 848	23.2	23.2
Secondary school (>7-10)	47 778	28.5	28.0	23 603	26.3	26.1
High Secondary or more (>10)	35 515	21.2	22.6	14 243	15.9	17.2
Mother can read local language	Missing = 1101			Missing = 679		
No	54 116	32.5	31.5	34 992	39.3	37.9
Yes	112 494	67.5	68.5	54 133	60.7	62.0
Preceding birth interval	Missing = 440			Missing = 230		
24 or more months or first child	141 583	84.6	84.4	74 090	82.7	82.4
Less than 24 mo	25 688	15.4	15.6	15 484	17.3	17.6
Maternal age at first childbirth	Missing = 4			Missing = 1		
<18 y old	20 482	12.2	12.9	12 094	13.5	14.0
18 y or older	147 225	87.8	87.1	77 709	86.5	86.0
Prenatal iron supplementation	Missing = 2750			Missing = 1330		
<3 mo	117 766	71.4	69.2	65 930	74.5	72.5
3 mo or more	47 195	28.6	30.8	22 544	25.5	27.5
Four or more antenatal care visits	Missing = 1598			Missing = 789		
No	86 784	52.2	48.4	50 826	57.1	53.3
Yes	79 329	47.8	51.6	38 189	42.9	46.7
**Household Factors**						
Below Poverty Line Card	Missing = 323			Missing = 145		
No	10 4501	62.4	61.6	53 073	59.2	58.8
Yes	62 887	37.6	38.4	36 586	40.8	41.2
Caste	Missing = 798			Missing = 456		
No Backward Caste	38 308	23.0	24.7	17 294	19.4	21.3
Other Backward Caste	64 750	38.8	43.6	35 721	40	43.6
Schedule Tribe	32 626	19.5	10.1	17 950	20.1	11.8
Scheduled Caste	31 229	18.7	21.6	18 383	20.6	23.3
Religion	Missing = 0			Missing = 0		
Hindu	126 919	75.7	81.3	69 424	77.3	81.5
Muslim	25 544	15.2	16.0	13 641	15.2	16.1
Christian	13 105	7.8	2.0	5600	6.2	1.7
Other	2143	1.3	0.6	1139	1.3	0.7
Toilet access	Missing = 0			Missing = 0		
No	104 135	62.1	44.2	59 673	66.5	51.6
Yes	63 550	37.9	55.7	30 117	33.5	48.4
Water treated before drinking	Missing = 26			Missing = 14		
No	92 198	61.9	66.9	53 417	69.8	70.0
Yes	56 730	38.1	33.1	26 931	30.2	30.0
Use soap to wash hands	Missing = 909			Missing = 501		
No	66 958	40.7	40.7	40 600	45.3	45.3
Yes	99 844	59.3	59.3	48 703	54.7	54.7
Finished floors	Missing = 0			Missing = 0		
No	85 861	51.2	47.6	51 572	57.4	53.8
Yes	81 850	48.8	52.4	38 232	42.6	46.2
Concrete walls	Missing = 0			Missing = 0		
No	55 119	32.9	25.9	32 496	36.2	29.6
Yes	112 592	67.1	74.1	57 308	63.8	70.5
Separate area for cooking	Missing = 65			Missing = 34		
No	58 373	34.8	43.2	35 068	39.1	48.9
Yes	109 273	65.2	56.8	54 702	60.9	51.1
Cooking fuel	Missing = 83			Missing = 47		
Solid fuel	112 936	67.4	62.6	66 131	73.7	69.2
Non-solid fuel	54 684	32.6	37.4	23 623	26.3	30.8
**Geographical factors:**						
Location	Missing = 0			Missing = 0		
Urban	42 093	25.1	28.9	19 710	21.9	25.4
Rural	125 618	74.9	71.1	70 094	78.1	74.6
State Focus	Missing = 0			Missing = 0		
Normal	42 676	25.4	43.5	21 450	23.9	39.9
Northeast	25 128	15	3.9	10 748	12	3.3
High	87 903	59.0	52.6	57 606	64.1	56.8
District Priority	Missing = 0			Missing = 0		
Normal	118 773	70.8	72.9	61 633	68.6	70.7
High	48 938	29.2	27.1	28 171	31.4	29.3
Average CIAF prevalence at district level	Missing = 0			Missing = 0		
20%-39.9%	11 815	7.0	4.8	3609	4.0	2.8
40%-49.9%	26 873	16	13.9	10 880	12.1	10.6
50%-59.9%	38 880	23.2	26.5	19 365	21.6	24.5
60%-69.9%	61 251	36.5	38.8	36 387	40.5	42.4
70%-79.9%	27 780	16.6	15.6	18 718	20.8	19.2
80%-89.9%	1112	0.7	0.4	845	0.9	0.3

CIAF – comprehensive indicator of anthropometric failure, w-% – weighted estimate of proportion

### Analysis

All statistical analyses were performed in STATA 15 MP using the svyset and svy commands to account for complex survey weighting of each participant. The associations between predictive variables and CIAF were evaluated using bivariate and multivariable logistic regression models to derive unadjusted and adjusted odds ratios. Individual predicted probabilities corresponding to unadjusted and adjusted models were calculated for analytic sample sets using the inverse logit transformation of beta coefficients for the participant’s covariate distribution. Each model’s discrimination ability was assessed by calculating c-statistic using “roctab” command for the predicted probabilities against the CIAF outcome. Similarly, model calibration was assessed using Brier scores and Hosmer-Lemeshow goodness of fit tables using decile grouping of predicted probabilities against the binary outcome variable. To achieve a parsimonious model, predictor variables were added in a stepwise manner and included in the model if their inclusion increased the full model’s multivariable c-statistic by at least 0.01 or reduced the brier score by at least 0.001. Continuous variables were categorized and the number of categories for existing discrete categories was minimized using receiver-operator curve analyses for multivariable models to achieve a model that is easy to implement in the real world. Birth weight was transformed to clinically meaningful categories of extremely low birth weight (<1800g), low birth weight (1800-2500g), or normal birth weight (>2500g). Maternal height was categorized into three categories (5′2” or taller, 4’8” to less than 5′2”, and less than 4’8”) by examining a plot of multivariable c-statistic against maternal height values to the nearest inch [25]. The final step examined interactions between variables in the fully specified model. Because inclusion of interaction variables did not improve model performance, they were not included in the final model.

Internal validation of the final model was performed using previously established methods to test and correct for optimism, which is the difference between the model’s c-statistic and the bias-corrected c-statistic of resampled data sets using nonparametric bootstrap methods [26,27]. A simulation study comparing internal validation performance of various methods found that the bootstrapping method outperformed various split-sample methods [28]. Therefore, a total of 200 data sets were resampled, representing participants corresponding to 14 000 PSU clusters selected with replacement. The final estimate of internal validity was derived by subtracting optimism from the model’s c-statistic to penalize the model for overfitting.

## RESULTS

**Table 1** describes the empirical and survey-weighted distribution of well-known predictors of child undernutrition among the children eligible for this analysis. More than half of the children (89 804; 53.6%) included in the analyses were undernourished based on the CIAF definition, corresponding to a weighted proportion of 54.4% (95% CI = 54.0%-54.8%). Three-fourths of all children surveyed lived in rural regions. More than half (54.0%) were male, and two-thirds had zero or one living sibling. Birth weight was available for 131 139 children and 37.0% weighed 2500g or less. The majority (83.7%) of the mothers of children included in the study had a stature shorter than 5 feet 2 inches. Nearly one in four mothers (28.5%) had no formal education and a third (32.5%) could not read their local language. Most children were from families that self-identified as belonging to an underprivileged caste (77%). Only 37.9% of children belonged to households that reported access to a toilet and two-thirds of the children belonged to families that use solid fuel for cooking. All predictors were closely associated with child undernutrition (*P* < 0.001).

After criteria-based model building and variable transformation, fifteen predictors, including three geographical variables, were selected in the final individual covariate model. The results of bivariate and multivariable logistic regression for the outcome of child undernutrition using these predictors are presented in **Table 2**. After adjusting for other covariates, all predictors remained strongly associated with the outcome of child undernutrition albeit the effect estimates were attenuated, with the exception of residence in high focus states or high priority districts. In bivariate regression, residence in a high focus state or high priority district increased the odds of child undernutrition. However, after accounting for other predictors, residence in these regions was associated with a moderate reduction in the odds of child undernutrition. Table S1 in the **Online Supplementary Document** describes the association of these factors with stunting as an outcome.

**Table 2:** Results of weighted logistic regression for the outcome of comprehensive index of anthropometric failure in the first five years of life based on data from 2015-2016 National Family Health Survey in India*

Risk Factors	Unadjusted			Adjusted			
**OR**	**LCI**	**UCI**	**β**	**OR**	**LCI**	**UCI**
**Maternal height: >5 feet 2 inches**	Ref			Ref			
4 feet 8 inches to less than 5 feet 2 inches	1.66	1.59	1.73	0.34	1.41	1.34	1.48
<4 feet 8 inches	2.99	2.84	3.14	0.79	2.20	2.07	2.33
**Maternal education: >10 y**	Ref			Ref			
No formal education	2.76	2.65	2.88	0.45	1.56	1.48	1.65
1-7 y of schooling	1.96	1.87	2.05	0.30	1.35	1.28	1.43
>7-10 y of schooling	1.46	1.40	1.52	0.17	1.19	1.13	1.25
**Sex: Female**	Ref			Ref			
Male	1.07	1.04	1.10	0.10	1.10	1.07	1.14
**Preceding birth interval >24 mo**	Ref			Ref			
≤ 24 mo	1.07	1.04	1.10	0.23	1.25	1.20	1.31
**Birthweight: >2500 g**	Ref			Ref			
<1800 g	2.63	2.40	2.87	0.88	2.42	2.20	2.66
1800-2500 g	1.72	1.66	1.78	0.46	1.59	1.53	1.65
**Number of Siblings: Zero or One**	Ref			Ref			
Two or more	1.48	1.43	1.54	0.07	1.07	1.03	1.11
**Low Caste: No**	Ref			Ref			
Yes	1.41	1.36	1.46	0.14	1.15	1.10	1.20
**Toilet access: Yes**	Ref			Ref			
No	1.95	1.89	2.00	0.18	1.20	1.15	1.25
**House at least partially finished: Yes**	Ref			Ref			
No	1.72	1.66	1.79	0.12	1.13	1.08	1.18
**Separate kitchen: Yes**	Ref			Ref			
No	1.48	1.43	1.53	0.07	1.07	1.03	1.12
**Cooking fuel: Non-solid Fuel**	Ref			Ref			
Solid fuel	1.77	1.71	1.83	0.07	1.08	1.03	1.12
**Uses soap after toilet use: Yes**	Ref			Ref			
No	1.52	1.47	1.56	0.07	1.08	1.04	1.12
**State Focus: Normal**	Ref			Ref			
Northeast Focus	0.89	0.85	0.94	-0.16	0.85	0.80	0.90
Other Focus	1.43	1.39	1.48	-0.23	0.80	0.76	0.83
**High Priority District: No**	Ref			Ref			
Yes	1.28	1.24	1.32	-0.09	0.92	0.88	0.95
**Mean district CIAF%: 20%-39.9%**	Ref			Ref			
40%-49.9%	1.54	1.41	1.69	0.29	1.33	1.21	1.47
50%-59.9%	2.22	2.04	2.41	0.60	1.83	1.67	2.01
60%-69.9%	3.21	2.96	3.49	0.87	2.40	2.18	2.63
70%-79.9%	4.46	4.10	4.86	1.11	3.03	2.75	3.35
80%-89.9%	7.51	5.85	9.65	1.64	5.16	3.88	6.86
**Constant**				-1.62			

OR – odds ratio, LCI – lower confidence interval, UCI – upper confidence interval, β – beta-coefficient, CIAF – composite index of anthropometric failure

*Multivariable c-statistic = 0.68 (optimism-corrected = 0.67) brier score = 0.225.

The final model had a reasonable discrimination ability as measured by a survey-weighted c-statistic of 0.68 (optimism-adjusted c-statistic: 0.67) and a Brier score of 0.225. The Hosmer-Lemeshow goodness of fit table for distribution of observed and expected prevalence for each decile risk group is presented in **Table 3**. One in four children categorized into the lowest risk group was undernourished while four in five children in the highest risk group were undernourished. The observed and expected prevalence were within 95% CI for each risk grouping. **Table 4** describes model performance and discrimination ability across different sub-groups. Overall, the model calculated the lowest individual probability of child undernutrition at 13.6% and highest probability at 92.0%. The model performed consistently across all subgroups except for children under the age of six months, for whom model had poor discrimination ability (c-statistic = 0.63).

**Table 3:** Hosmer-Lemeshow goodness of fit table for distribution of observed CIAF prevalence vs predicted prevalence across decile risk groups for undernutrition among the children under the age of five from the 2015-2016 National Family Health Survey in India

Decile Risk	N	Observed CIAF%	Observed-weighted CIAF%	Predicted CIAF%
1	12 924	25.1	25.9 (24.5-27.3)	25.4
2	12 884	34.1	34.5 (33.1-36.0)	33.5
3	12 932	39.2	37.9 (36.6-39.2)	39.1
4	12 878	44.3	44.1 (42.8-45.4)	44.2
5	12 909	49.7	49.2 (47.9-50.6)	48.9
6	12 897	53.8	53.1 (51.9-54.3)	53.6
7	12 907	58.9	57.9 (56.8-59.0)	58.2
8	12 902	63.3	63.0 (61.8-64.1)	63.1
9	12 903	69.1	69.1 (68.1-70.1)	68.7
10	12 904	77.6	77.3 (76.4-78.2)	77.0

CIAF – composite index of anthropometric failure

**Table 4:** Model performance and discrimination ability across different sub-groups

Group	N	Observed CIAF%	Predicted CIAF%	Predicted Min Risk	Predicted Max Risk	c-statistic
**Overall**	129 040	51.5	51.1	13.6	92.0	0.68
**Child Age**						
0-5 mo	14 622	50.1	51.8	13.6	91.9	0.63
6-24 mo	53 579	51.9	51.8	13.6	91.9	0.69
>24 mo	60 839	51.5	50.3	13.6	92.0	0.69
**Location**						
Rural	92 693	53.9	53.5	13.6	92.0	0.68
Urban	36 347	45.5	45.2	13.6	91.0	0.66
**State Focus**						
Normal	39 565	49.4	49.0	15.4	92.0	0.66
Northeast	17 766	40.0	40.3	14.5	84.7	0.66
High	71 709	55.5	55.0	13.6	91.9	0.68
**District Priority**						
Yes	33 514	55.5	54.3	13.8	92.0	0.68
No	95 526	50.1	50.0	13.6	91.9	0.67

CIAF – composite index of anthropometric failure

## DISCUSSION

In this study, we used data from a nationally representative survey to develop a predictive algorithm that can predict five-year risk of undernutrition among Indian children at the time of their delivery. The model uses information about the child, child’s mother, child’s household, and the child’s geographical region. All factors included in the final model have been identified as closely associated with child undernutrition in previous studies using data from the 2004-2005 National Family Health Surveys [22-24]. It is important to note that the factors included in the model do not represent a comprehensive list of predictors of child undernutrition in India, but rather those that can be collected at the time of delivery or in a reasonable time frame. For instance, breastfeeding practices and timely introduction of complementary foods play an important role in child nutrition but cannot be captured at the time of the delivery. This approach lowers the performance of a predictive algorithm but allows for identification of at-risk children at the time of their birth and can empower FHWs to make informed decisions for prioritizing services and surveillance of the vulnerable children.

Health Information Systems play an important role in facilitating routine service delivery activities by the FHWs. India launched the Mother and Child Tracking System (MCTS) in 2009 as a web-based portal that collected data from FHWs for all pregnant women in their region, especially at the time of delivery [29]. The MCTS generates automated schedules for FHW for services due and sends SMS reminders to FHW and the beneficiaries, ensuring continued medical care. Evaluation of the MCTS has demonstrated its benefits in helping FHW more effectively provide services and follow-up with mother and young children in their region [30,31]. As technological capacity advances, health information systems such as MCTS can be used to facilitate targeting the most vulnerable populations. Integration of the model developed in our study within the MCTS presents such an opportunity to employ data-driven approaches for improving decision making by FHW, supervisory, and managerial health officials. Because ten of the fifteen questions included in our predictive model are already captured by the MCTS, the additional burden of data collection for FHW (toilet access, living in semi-finished or finished house, type of cooking fuel, using soap after toilet use) will be minimal if the predictive model was integrated as part of the MCTS [29].

The high prevalence of child undernutrition and the limited discrimination ability of the model dictates a careful use of the risk score. Intensification of resources for high-risk groups should be favoured over withdrawal of resources from low-risk groups. One approach could be to use the risk score to inform the frequency of counselling and follow-up from FHW. A Bangladeshi program focused on a population of 8.5 million mothers led to rapid and significant improvements in key breastfeeding and complementary feeding practices because of promotion strategies that targeted high priority groups through more frequent contacts [32]. The program found that complementing mass media campaigns with innovative approaches to improve the performance of FHW in delivering timely counselling to high-risk mothers were central to its success. Thus, an automatically generated schedule for children based on their risk score can ease the burden on FHW and allow them to provide services and monitor child growth in a timely manner.

In addition to helping the FHW risk-stratify and efficiently provide services for the most vulnerable children, the results of the model can also inform priority setting and resource allocation at the district, state, and national levels. Currently, the Indian government prioritizes funding allocation for implementation of national level programs for high-focus states and high-priority districts [14-17]. Our results show that the children living in these regions were more likely to be undernourished, but after accounting for other risk factors, they had lower odds of being undernourished than a child with the same covariate pattern living in a normal-focus state or priority district. A possible explanation for this discrepancy might be that the added resources allocated to high-priority regions helps prevent undernutrition among vulnerable children in comparison to their counterparts in other regions. Therefore, allocating resources based on regional distribution of risk-score might provide a more equitable approach.

It is important to consider certain limitations of our study. This model is based on self-reported data collected as part of a cross-sectional survey study. Although the survey was conducted by research staff, who received rigorous training in administering standardized questionnaires, recall bias on behalf of the respondents cannot be ruled out. However, the final model includes objective questions that are less prone to such bias. The use of an indicator variable (CIAF) likely leads to lack of precision and negatively impacts the performance of the model; however, we favour this approach, because CIAF in early childhood is associated with meaningful physical and cognitive outcomes [33]. An underlying assumption of the model is that the responses about household characteristics, ie, access to toilet, treatment of drinkable water, use of cooking fuel do not change throughout the first five years. It is plausible that water and sanitation hygiene improved after childbirth, especially if the child experienced illness or was undernourished. In this scenario, the coefficient of associations presented in our analyses are likely an underestimation. Due to the cross-sectional nature of the survey, we cannot test this assumption. Our final model is derived from nationally representative data, was internally validated using 200 random bootstrapped samples, and had reasonable predictive capability. However, further work is necessary to externally assess and validate this model and its performance in real-world settings.

One such opportunity comes from its integration within the existing MCTS portal. The additional data on feeding practices, immunization records, and growth outcomes as the child advances in age can be used to augment the model and calculate a dynamic risk-score for child undernutrition that changes over time. By comparing the risk score with the outcome of child undernutrition in the first five years, the model can be calibrated further using decision curve analyses to identify risk thresholds for child undernutrition. Thus, the current model developed by this study represents a first step in adopting a risk-score based approach for the most vulnerable population to receive services in a timely manner.

## CONCLUSION

This article describes the development and validation of a predictive algorithm to identify newborn's risk of developing undernutrition in the first five years of life using data that is routinely available at the time of delivery. This approach can facilitate efficient allocation of scarce resources, especially when leveraged with existing public health infrastructure.

## Additional material


Online Supplementary Document
