# Supplementary material for: Predictive algorithm to stratify newborns at-risk for child undernutrition in India: Secondary analysis of the National Family Health Survey-4
Source: J Glob Health. 2022 May 14;12:04040. doi: 10.7189/jogh.12.04040 (PMC9107290; doi:10.7189/jogh.12.04040)
Supplement: Online Supplementary Document [file jogh-12-04040-s001.pdf]

Table S1: Results of weighted logistic regression for the outcome of Comprehensive Index of Anthropometric Failure (CIAF) and stunting in the first 5 years of life based on data from 2015-16 National Family Health Survey in India						
Risk Factors	CIAF			Stunting		
	OR	LCI	UCI	OR	LCI	UCI
**Maternal height: > 5’2”**	Ref					
4’8” to < 5’2”	1.41	1.34	1.48	1.58	1.49	1.67
< 4’8”	2.20	2.07	2.33	2.62	2.45	2.81
**Maternal education: > 10 years**	Ref					
No formal education	1.56	1.48	1.65	1.72	1.61	1.84
1-7 years of schooling	1.35	1.28	1.43	1.44	1.34	1.53
>7-10 years of schooling	1.19	1.13	1.25	1.25	1.18	1.32
**Sex: Female**	Ref					
Male	1.10	1.07	1.14	1.15	1.11	1.20
**Birth interval > 24 months**	Ref					
<= 24 months	1.25	1.20	1.31	1.35	1.28	1.42
**Birthweight: > 2500 grams**	Ref					
< 1800 grams	2.42	2.20	2.66	2.04	1.84	1.32
1800 – 2500 grams	1.59	1.53	1.65	1.43	1.37	1.49
**# Siblings: Zero or One**	Ref					
Two or more	1.07	1.03	1.11	1.14	1.09	1.21
**Low Caste: No**	Ref					
Yes	1.15	1.10	1.20	1.15	1.09	1.21
**Toilet access: Yes**	Ref					
No	1.20	1.15	1.25	1.16	1.10	1.21
**Partially finished House: Yes**	Ref					
No	1.13	1.08	1.18	1.02	0.97	1.07
**Separate kitchen: Yes**	Ref					

No	1.07	1.03	1.12	1.05	1.01	1.10
**Cooking fuel: Non-solid Fuel**	Ref					
Solid fuel	1.08	1.03	1.12	1.13	1.07	1.18
**Uses soap after toilet use: Yes**	Ref					
No	1.08	1.04	1.12	1.04	1.00	1.08
**State Focus: Normal**	Ref					
Northeast Focus	0.85	0.80	0.90	0.95	0.88	1.02
Other Focus	0.80	0.76	0.83	0.89	0.84	0.93
**High Priority District: No**	Ref					
Yes	0.92	0.88	0.95	0.98	0.93	1.02
**District CIAF%: 20 – 39.9%**	Ref					
40 – 49.9%	1.33	1.21	1.47	1.48	0.78	2.81
50 – 59.9%	1.83	1.67	2.01	1.68	0.89	3.17
60 – 69.9%	2.40	2.18	2.63	2.16	1.15	4.07
70 – 79.9%	3.03	2.75	3.35	2.86	1.52	5.40
80 – 89.9%	5.16	3.88	6.86	3.75	1.90	7.40
Multivariable c-statistic	0.68			0.67		
